# Renal function after out-of-hospital cardiac arrest; the influence of temperature management and coronary angiography, a post hoc study of the target temperature management trial

**DOI:** 10.1186/s13054-019-2390-0

**Published:** 2019-05-08

**Authors:** Malin Rundgren, Susann Ullén, Matt P. G. Morgan, Guy Glover, Julius Cranshaw, Nawaf Al-Subaie, Andrew Walden, Michael Joannidis, Marlies Ostermann, Josef Dankiewicz, Niklas Nielsen, Matthew P. Wise

**Affiliations:** 1 Department of Clinical Sciences, Anaesthesia and Intensive Care, Skane University Hospital, Lund University, 221 85 Lund, Sweden; 2 Foprum South, Skane University Hospital, Lund, Sweden; 3 Honorary Research Fellow, Cardiff University School of Medicine, Cardiff, UK; 4 Department of Intensive Care, Guys and St Thomas’ Hospital, Kings College London, London, UK; 5 Department of Anaesthetics and Intensive Care Medicine, Royal Bournemouth Hospital, Bournemouth, UK; 6 Adult Intensive Care Directorate, St George’s Hospital London, London, UK; 7 Department of Intensive Care Medicine, Royal Berkshire Hospital, Reading, UK; 8 Division of Intensive Care and Emergency Medicine, Department of Internal Medicine, Medical University Innsbruck, Innsbruck, Austria; 9 Department of Critical Care and Nephrology, Guy’s and St Thomas’ Hospital, King’s College London, London, UK; 10 Department of Cardiology, Skane University Hospital, Lund University, Lund, Sweden; 11 Department of Anaesthesia and Intensive Care, Helsingborg Hospital, Helsingborg, Sweden; 12 Adult Critical Care, University Hospital of Wales, Cardiff, UK; 13 Department of Intensive and Perioperative Care, Skane University Hospital, Lund University, 221 85 Lund, Sweden

**Keywords:** Out-of-hospital cardiac arrest, Acute kidney injury, Angiography, Induced hypothermia (target temperature management), Contrast

## Abstract

**Background:**

To elucidate the incidence of acute kidney injury (AKI) after out-of-hospital cardiac arrest (OHCA) and to examine the impact of target temperature management (TTM) and early coronary angiography on renal function.

**Methods:**

Post hoc analysis of the TTM trial, a multinational randomised controlled trial comparing target temperature of 33 °C versus 36 °C in patients with return of spontaneous circulation after OHCA. The impact of TTM and early angiography (within 6 h of OHCA) versus late or no angiography on the development of AKI during the 7-day period after OHCA was analysed. AKI was defined according to modified KDIGO criteria in patients surviving beyond day 2 after OHCA.

**Results:**

Following exclusions, 853 of 939 patients enrolled in the main trial were analysed. Unadjusted analysis showed that significantly more patients in the 33 °C group had AKI compared to the 36 °C group [211/431 (49%) versus 170/422 (40%) *p* = 0.01], with a worse severity (*p* = 0.018). After multivariable adjustment, the difference was not significant (odds ratio 0.75, 95% confidence interval 0.54–1.06, *p* = 0.10].

Five hundred seventeen patients underwent early coronary angiography. Although the unadjusted analysis showed less AKI and less severe AKI in patients who underwent early angiography compared to patients with late or no angiography, in adjusted analyses, early angiography was not an independent risk factor for AKI (odds ratio 0.73, 95% confidence interval 0.50–1.05, *p* = 0.09).

**Conclusions:**

In OHCA survivors, TTM at 33 °C compared to management at 36 °C did not show different rates of AKI and early angiography was not associated with an increased risk of AKI.

**Trial registration:**

NCT01020916. Registered on www.ClinicalTrials.gov 26 November 2009 (main trial).

**Electronic supplementary material:**

The online version of this article (10.1186/s13054-019-2390-0) contains supplementary material, which is available to authorized users.

## Background

Cardiac arrest (CA) with a return of spontaneous circulation (ROSC) represents a multisystem insult. Although neurological injury accounts for the majority of deaths, refractory cardiovascular failure is common. The post cardiac arrest syndrome is characterised by multi-organ dysfunction as a result of an acute inflammatory response and persistent haemodynamic instability. Ischaemia-reperfusion injury and on-going shock may aggravate renal injury [1–3]. Acute kidney injury (AKI) is common in these circumstances, affecting 12–81% of patients depending on definition and patient selection [1, 2, 4, 5]. It is possible that routine therapeutic interventions and diagnostic procedures, such as target temperature management (TTM) and scans with contrast, impact the development of organ dysfunction, including the development of AKI.

There are limited data on the renal effect of induced hypothermia and TTM after CA with ROSC. In the hypothermia after cardiac arrest study [6], patients randomised to treatment at 33 °C had a lower calculated glomerular filtration rate compared to controls [7]. In other clinical circumstances, hypothermia and rewarming had mixed effects [8, 9]. Prolonged hypothermia after coronary artery bypass surgery did not confer renoprotection, and relatively rapid rewarming to normothermia during cardiopulmonary bypass was associated with more renal dysfunction than less aggressive rewarming [10].

The acute coronary syndrome is a common cause of CA. Current recommendations for ST-elevation myocardial infarction (STEMI) after CA with ROSC include emergent coronary angiography, and if necessary, percutaneous coronary intervention (PCI) within 120 min [11]. However, in comatose CA patients after non-STEMI, the decision for angiography and the optimal timing remains controversial [11, 12]. Trials are addressing the risk-benefit ratio of emergent coronary angiography in this patient group [13, 14]. Whether early angiography impacts the risk of AKI after CA is unknown.

The aims of this post hoc analysis were to describe the incidence and outcome of AKI in survivors of out-of-hospital CA (OHCA) of presumed cardiac origin and to investigate the impact of TTM and early coronary angiography.

## Methods

We performed a post hoc analysis of the target temperature management trial, a parallel group randomised clinical trial in 36 intensive care units (ICUs) in Europe and Australia between 2010 and 2013 which compared the effect of treatment at 33 °C or 36 °C following ROSC on all-cause mortality (trial registration number NCT01020916, registered on www.ClinicalTrials.gov 26 November 2009). The rationale and main results have already been reported [15]. Ethics committees in all participating countries approved the protocol. Waived, delayed and/or consent from legal surrogates and delayed written informed consent from patients regaining mental capacity were obtained according to decisions by ethical boards in respective countries.

### Patient population

Comatose adult patients with ROSC after OHCA of the presumed cardiac cause were eligible for inclusion. Patients were excluded if the time between ROSC and screening exceeded 4 h, CA was unwitnessed with asystole as first recorded rhythm, intracranial bleeding or ischaemic stroke was suspected or initial temperature on admission was less than 30 °C. Sustained ROSC was defined as no requirement for chest compressions for 20 consecutive minutes and persisting signs of circulation, including systolic blood pressure (BP) > 80 mmHg with or without vasoactive drugs. The time of ROSC was defined as the start of sustained ROSC [16]. Shock on admission was defined as need for vasopressors, intra-aortic balloon pump counterpulsation (IABP) or other mechanical support to maintain a systolic BP > 80 mmHg for > 30 min after ROSC.

### Randomisation, intervention and post cardiac arrest care

Randomisation was stratified by the centre in a 1:1 ratio between TTM at 33 °C (TTM-33) and 36 °C (TTM-36) and temperature controlled using commercially available cooling devices. Centres were encouraged to facilitate cooling by using 30 mL/kg of crystalloid fluid at cold or room temperature, depending on temperature allocation. Target temperature was induced and maintained for 28 h after ROSC, followed by controlled rewarming at a maximum rate of 0.5 °C/h. In the absence of pre-specified criteria, withdrawal of life-supporting treatment was not allowed prior to 108 h after randomisation [17]. Other management decisions were at the discretion of the clinicians responsible for the patient.

### General data collection

The following data were collected from the TTM database: demographics, pre-hospital factors, concurrent diseases, status on arrival in hospital, Sequential Organ Failure Assessment (SOFA) score, serum creatinine, urine output, fluid balance, level of inotropic/vasoactive support and use of renal replacement therapy (RRT) on a daily basis until day 7 or discharge from ICU (whichever occurred first), ICU and hospital length of stay and survival status at discharge and 6 months after CA.

### Selection of patients for analysis of AKI after CA

To assess the development of AKI, patients surviving beyond day 2 after CA were included. Patients with end-stage renal failure on chronic dialysis before CA were excluded. In the first analysis, the development of AKI was stratified by temperature allocation. In a second analysis, patients undergoing early coronary angiography within 6 h of CA were compared with patients who had delayed or no angiography. The 6-h time limit defining early angiography in the study was chosen to allow time for transport and primary resuscitation in case of haemodynamic instability. If the time of angiography was not available, patients were excluded from the angiography analysis.

### Renal function assessment

AKI was defined according to the KDIGO criteria [18] with modifications. The analysis included the ICU length of stay up to a maximum of 7 days. For the creatinine-based criteria, we used the first recorded creatinine after arrival in the hospital as the baseline value. Daily serum creatinine was measured locally in hospital laboratories or using point-of-care devices. As only total daily urine output data on individual days were recorded in the case report forms, a mean hourly urine rate per day was calculated and adjusted for the recorded body weight. For this reason, we applied the urine criteria of the KDIGO classification as follows: AKI stage 2 was defined by a mean urine production < 0.5 mL/kg/h for the whole 24-h period, and AKI stage 3 was defined by a mean urine production < 0.3 mL/kg/h for the whole 24-h period. The urine criterion was not used to define AKI stage 1. For a summary of definitions, see Additional file 1: Table S1.

Patients were included in the study at any time of day. As such, the 24-h urine output and fluid balance data were incomplete on the day of CA (day 1) and also potentially on the last day in ICU. To avoid misdiagnosing patients as having AKI based on a potentially erroneous low urine output, data from incomplete days were excluded from the analysis. The urine output on the last day of ICU stay was disregarded if the patient stayed less than 7 whole ICU days.

The worst stage of AKI based on serum creatinine, urine output or initiation of RRT was determined on a daily basis.

### Statistical analysis

Data are presented as counts and percentages or, for continuous data, as mean (standard deviation) or median [interquartile range (IQR)] depending on distribution. Between-group comparisons were made using chi-squared or Mann-Whitney *U* tests. Logistic regression was used to assess the impact of TTM and early coronary angiography on AKI. The model was adjusted for patient factors and previous comorbidities [age, sex, hypertension, diabetes, New York Heart Association (NYHA) heart failure class 3 or 4 and serum creatinine on admission], cardiac arrest circumstances [applications of bystander cardiopulmonary resuscitation (CPR), shockable rhythm, time from CA to ROSC] and post cardiac arrest factors (lactate clearance defined as a reduction of lactate > 50% during the first 12 h or lactate < 2 mmol/L at 12 h after CA, use of vasopressors and/or inotropes, IABP insertion, highest blood glucose during the first 24 h, temperature allocation and angiography within 6 h of CA). As a secondary analysis, a logistic regression was performed to address the impact of angiography within 36 h after the CA on AKI. *P* < 0.05 was considered significant. No corrections for multiple comparisons were made. The IBM SPSS Statistics for Windows, Version 22 (IBM Corp. Armonk, NY) was used for the required analyses.

## Results

Nine hundred thirty-nine patients were included in the original TTM study. Following the exclusion of 86 patients, 853 patients were included in the analyses (Additional file 1: Figure S1). The patients excluded due to early death survived 15 (IQR 10–21) hours after CA. Seven hundred six patients (82%) were males, and the median age was 65 (56–73) years. An initial shockable rhythm was present in 80% with bystander CPR performed in 625/853 patients (73%). The median time from CA to ROSC was 25 (16–38) minutes. More than 80% of the patients required vasoactive drugs during the first 3 days of intensive care and 112/859 (13%) were in shock at the time of inclusion in the study. More than half of the patients had a maximum circulatory SOFA score of 4 during the first 3 days after CA (Table 1).

**Table 1:** Patient characteristics split into no AKI or any AKI

Baseline variables	No AKI (*n* = 472)	Any AKI (*n* = 381)	*p* value
Age (years)	63 (54–72)	66 (59–74)	0.0001
Sex (male)	374 (79%)	321/381 (84%)	0.06
Body mass index	25.3 (23.3–27.8)	26.3 (24.2–29.7)	< 0.0001
Hypertension	179/471 (38%)	162/379 (43%)	0.18
CHF (NYHA 3–4)	23/472 (5%)	33/379 (9%)	0.03
Any IHD, PCI, AMI, CABG	137/472 (29%)	129/377 (32%)	0.12
Diabetes	63/469 (13%)	58/379 (15%)	0.49
Cardiac arrest variables			
Shockable rhythm	403/472 (85%)	283/381 (74%)	< 0.0001
Bystander CPR	357/472 (76%)	268/381 (70%)	0.09
Time CA-ROSC (min)	23 (15–33)	29 (19–41)	< 0.0001
Shock on admission	41/472 (9%)	71/381 (19%)	0.0001
Lactate on admission (mmol/L)	5.3 (2.7–8.4) (*n* = 437)	6.2 (3.0–9.8) (*n* = 362)	0.004
IABP	52/472 (11%)	84/381 (21%)	< 0.0001
TTM 33 °C	220/472 (47%)	211/381 (55%)	0.01
Early angiography	301/459 (66%)	215/376 (57%)	0.01
Any vasoactive drug day 1	357/467 (76%)	292/378 (77%)	0.81
Day 2	384/466 (82%)	321/377 (85%)	0.30
Day 3	311/466 (67%)	301/377 (80%)	< 0.0001
Noradrenaline or epinephrine > 0.1 μg/kg/min days 1–3	207/472 (44%)	233/381 (61%)	< 0.0001
Renal variables/outcomes			
Baseline creatinine (μmol/L)	100 (80–115) (*n* = 455)	110 (90–135) (*n* = 372)	< 0.0001
Creatinine > 130 μmol/L	51/455 (11%)	99/372 (27%)	< 0.0001
Worst AKI stage in the first week			
Stage 1	NA	138/381 (36%)	NA
Stage 2	NA	116/381 (30%)	NA
Stage 3	NA	127/381 (33%)	NA
Daily fluid balance			
Day 2	1300 (400–2300) (*n* = 467)	1800 (800–3100) (*n* = 381)	< 0.0001
Day 3	400 (− 700 to + 1100) (*n* = 454)	700 (− 100 to + 2000) (*n* = 376)	< 0.0001
RRT during the first week	NA	74/381 (20%)	NA
Survival at 6 months	317/472 (67%)	159/381 (42%)	< 0.0001

Patient characteristics, cardiac arrest-related factors, treatment and renal outcome data split into AKI or no AKI during days 2–7 of ICU stay

*Abbreviations: CHF* chronic heart failure, *IHD* ischaemic heart disease, *PCI* percutaneous coronary intervention, *AMI* acute myocardial infarction, *CABG* coronary artery bypass grafting, *CPR* cardiopulmonary resuscitation, *CA-ROSC* time from cardiac arrest to return of spontaneous circulation, *IABP* intra-aortic balloon pump, *TTM* target temperature management, *RRT* renal replacement therapy, *NA* not applicable

**Fig. 1:**
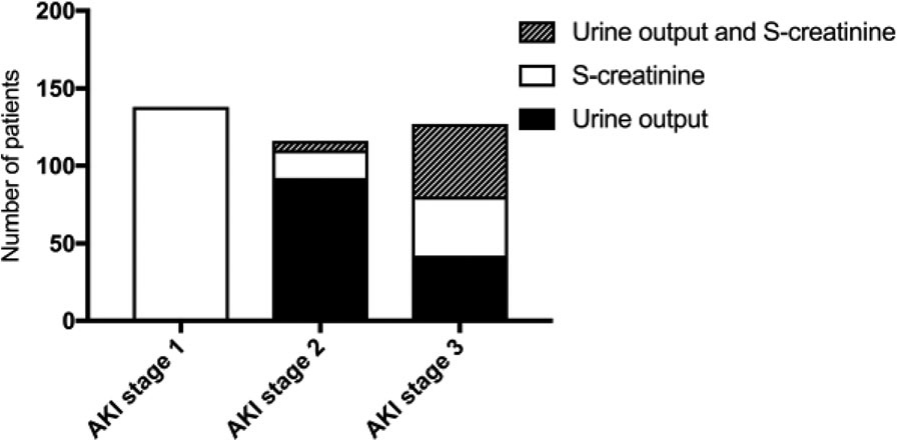
The frequency of criteria used to determine the worst stage of AKI days 2–7 after CA. AKI stages 2 and 3 are split into the different diagnostic criteria urine output, S-creatinine concentration or a combination thereof. For stage 1, AKI urine output criterion was by study definition not applicable and as a consequence neither was the combination of urine output and creatinine level

During days 2–7, 138 patients (16%) had maximum AKI stage 1, 116 (14%) had AKI stage 2 and 127 (15%) had AKI stage 3 of whom 74 received RRT. There were 472 patients (55%) who did not fulfil the AKI criteria. The daily prevalence of AKI during the first week is shown in Additional file 1: Figure S2. The number of patients diagnosed with AKI based on creatinine and urinary output criteria is shown in Fig. 1. In 541 patients (264 TTM-33 and 277 TTM-36), their last day of the ICU stay was incomplete because the patient left the ICU either due to improvement or death/withdrawal of intensive care. Urine output analysis was therefore excluded for that day.

Patients who developed AKI were characterised by an older age, a greater proportion suffering from NYHA heart failure stages 3 and 4, a lower incidence of a shockable rhythm, a longer duration from CA to ROSC, a higher lactate and serum creatinine on admission and more frequent treatment with an IABP, while early angiography and TTM-36 were associated with less AKI (Table 1).

### Impact of TTM

**Fig. 2:**
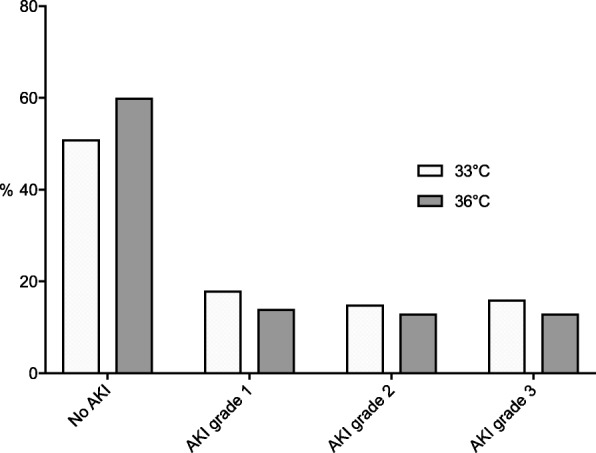
The maximum stage of AKI during days 2–7 after cardiac arrest stratified by temperature allocation. The category of AKI was worse in the TTM-33 group (*p* = 0.018, Mann-Whitney *U* test)

In the unadjusted analysis, AKI was more common in patients randomised to TTM-33 compared to TTM-36 (49% versus 40%, *p* = 0.01). The severity of AKI was also worse in the TTM-33 group (*p* = 0.018, Fig. 2), but there was no difference in the proportion of patients who received RRT. The TTM-33 group had a significantly more positive fluid balance, and more patients required vasoactive drugs during days 2–3 (Additional file 1: Table S2). Fifty-six percent of patients in the TTM-33 group and 47% in the TTM-36 group had a cardiovascular SOFA score of 4 on at least 1 day during days 1–3 (*p* = 0.45).

Logistic regression analysis with correction for co-morbidities and factors related to CA and critical care demonstrated that TTM-36 was not an independent risk factor for AKI (*p* = 0.10, odds ratio (OR) 0.75, 95% confidence interval (CI) 0.54–1.06) (Table 2).

**Table 2:** Results of logistic regression

	Significance	Odds ratio	95% CI
Coronary angiography < 6 h	0.09	0.73	0.50–1.05
Temperature (36 °C)	0.10	0,75	0.54–1.06
Age (per year)	0.18	1.01	0.99–1.03
Sex (female)	0.09	0.67	0.43–1.06
Diabetes	0.07	0.61	0.36–1.04
Hypertension	0.60	1.10	0.77–1.58
Heart failure	0.50	1.26	0.64–2.46
Creatinine on admission	0.02	1.01	1.00–1.01
Time to ROSC (per minute)	0.05	1.01	1.00–1.02
Bystander CPR	0.62	1.10	0.74–1.63
Shock	0.18	1.42	0.84–2.41
Shockable rhythm	0.04	0.63	0.40–0.98
Lactate clearance	0.20	0.77	0.52–1.14
No vasopressor, dobutamine or DA < 5 μg/kg/min	0.32		
DA > 5 μg/kg/min or NE < 0.25 μg/kg/min	0.52	0.87	0.56–1.34
NE or Epi ≥ 0.25 μg/kg/min	0.30	1.34	0.77–2.35
NE or Epi ≥ 0.75 μg/kg/min	0.67	1.23	0.43–3.47
IABP	0.003	2.22	1.32–3.74
Maximum blood glucose within 24 h	0.24	1.02	0.98–1.06

Results of logistic regression, angiography within 6 h of cardiac arrest vs. no early angiography. Increasing time to ROSC, a higher serum creatinine on admission and treatment with IABP were independently associated with a higher risk of AKI whereas an initial shockable rhythm was associated with less AKI. Normal lactate clearance was defined as lactate < 2.0 mmol/L alt 12 h or a decrease in lactate > 50% within 12 h of cardiac arrest

*Abbreviations: ROSC* return of spontaneous circulation, *CPR* cardiopulmonary resuscitation, *DA* dopamine, *NE* norepinephrine, *Epi* epinephrine, *IABP* intra-aortic balloon pump

### Impact of early coronary angiography

**Fig. 3:**
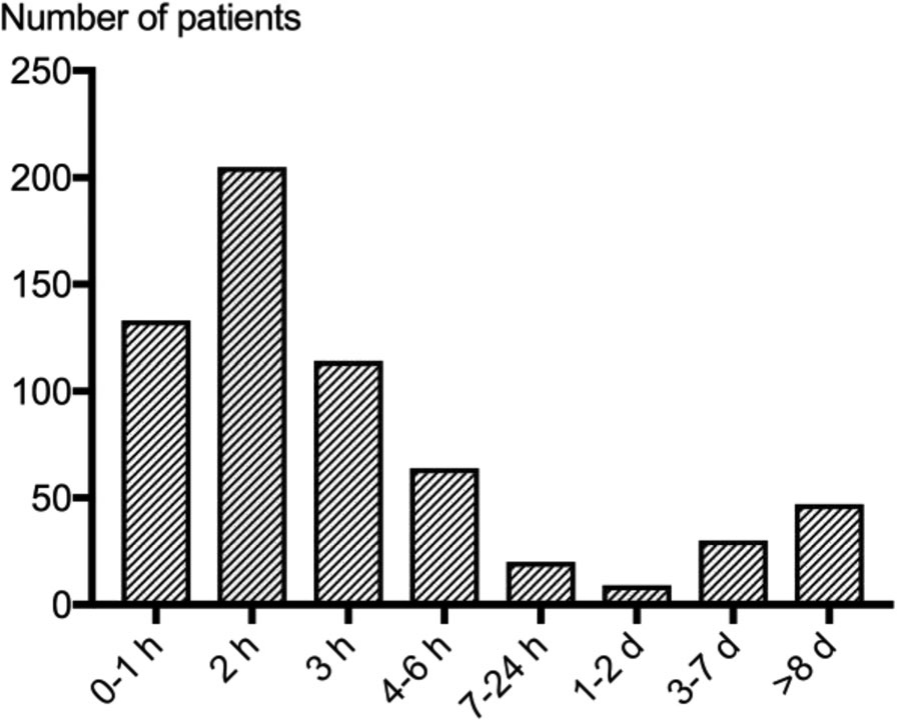
The time from cardiac arrest to angiography. The majority of patients had an early angiography (within 6 h of cardiac arrest). Note varied time intervals

Early angiography within 6 h of CA was performed in 516/836 patients (62%) (Table 3). The median time between CA and angiography was 2 (1–3) hours (Fig. 3). Two hundred sixteen over five hundred seventeen (42%) of patients who underwent early angiography developed AKI compared to 161/319 (50%) of patients who had an angiogram later than 6 h following OHCA or not at all (*p* = 0.014). The severity of AKI was worse in patients without early angiography (*p* = 0.007). This difference was driven by patients not receiving any angiography. Logistic regression analysis demonstrated that increasing time to ROSC, a higher serum creatinine on admission and treatment with IABP were independently associated with a higher risk of AKI whereas an initial shockable rhythm was associated with less AKI. Timing of angiography was not an independent risk factor (*p* = 0.09) (Table 3). There were 128 patients in the late/no angiography group who underwent angiography more than 6 h after the CA (range 7 h–68 days after CA, Additional file 1, Fig. 3). A secondary analysis of angiography prior to 36 h did not alter the results.

**Table 3:** Patient characteristics split into angiography within 6 h of CA or later/no angiography

	Coronary angiography in the first 6 h (*n* = 516)	No coronary angiography in first 6 h (*n* = 319)	*p* value
Baseline variables			
Age (years)	63 (56–70)	69 (59–77)	< 0.0001
Sex (male)	438/516 (85%)	247/319 (77%)	0.01
Hypertension	220/514 (43%)	118/319 (37%)	0.11
CHF (NYHA 3–4)	25/515 (5%)	31/319 (10%)	0.01
Any IHD, PCI, AMI, CABG	149/516 (29%)	118/319 (37%)	0.02
Diabetes	62/515 (12%)	59/316 (19%)	0.01
Cardiac arrest variables			
Shockable rhythm	438/516 (84%)	231/319 (73%)	< 0.0001
Bystander CPR	393/516 (76%)	223/319 (70%)	0.05
Time CA-ROSC (min)	25 (17–38)	25 (16–40)	0.85
Shock on admission	67/516 (13%)	44/319 (14%)	0.75
Lactate on admission (mmol/L)	5.1 (2.4–8.8) (*n* = 477)	6.2 (3.6–9.5) (*n* = 310)	0.001
ST-elevation on ECG	279/513 (54%)	57/315 (18%)	< 0.0001
PCI	342/516 (66%)	23/319 (7%)	< 0.0001
IABP	107/515 (20%)	25/319 (8%)	< 0.0001
TTM 33 °C	254/516 (49%)	157/319 (49%)	1.00
Any vasoactive drug day 1	404/511 (79%)	233/317 (74%)	0.07
Day 2	438/510 (86%)	252/316 (80%)	0.03
Day 3	377/508 (74%)	221/317 (70%)	0.17
Noradrenaline or epinephrine > 0.1 μg/kg/min days 1–3	261/516 (50%)	171/317 (54%)	0.35
Renal variables/outcomes			
Baseline creatinine (μmol/L)	100 (85–120) (*n* = 499)	105 (85–130) (*n* = 312)	0.04
Creatinine > 130 μmol/L	78/499 (16%)	68/312 (22%)	0.03
Worst AKI stage first week			
Stage 1	86/516 (17%)	51/319 (16%)	0.85
Stage 2	59/516 (11%)	55/319 (17%)	0.02
Stage 3	71/516 (14%)	55/319 (17%)	0.20
Any AKI first week	216/516 (42%)	161/319 (50%)	0.02
Daily fluid balance			
Day 2	1500 (500–2500) (*n* = 513)	1600 (500–2700) (*n* = 318)	0.64
Day 3	300 (− 500 to + 1400) (*n* = 504)	500 (− 400 to + 1800) (*n* = 309)	0.12
RRT during first week	47/516 (9%)	27/319 (9%)	0.80
Survival at 6 months	311/516 (60%)	153/319 (48%)	0.0006

Patient characteristics, cardiac arrest-related factors, treatment and renal outcome data split into patients exposed to angiography within the first 6 h of cardiac arrest or not. Dichotomous results are presented as numbers and percentages; continuous data are presented as median and interquartile range. The *p* values were calculated using Fisher’s exact test and Mann-Whitney test respectively

*Abbreviations: CHF* chronic heart failure, *IHD* ischaemic heart disease, *PCI* percutaneous coronary intervention, *AMI* acute myocardial infarction, *CABG* coronary artery bypass grafting, *CPR* cardiopulmonary resuscitation, *CA-ROSC* time from cardiac arrest to return of spontaneous circulation, *IABP* intra-aortic balloon pump, *TTM* target temperature management, *RRT* renal replacement therapy

### Mortality

**Fig. 4:**
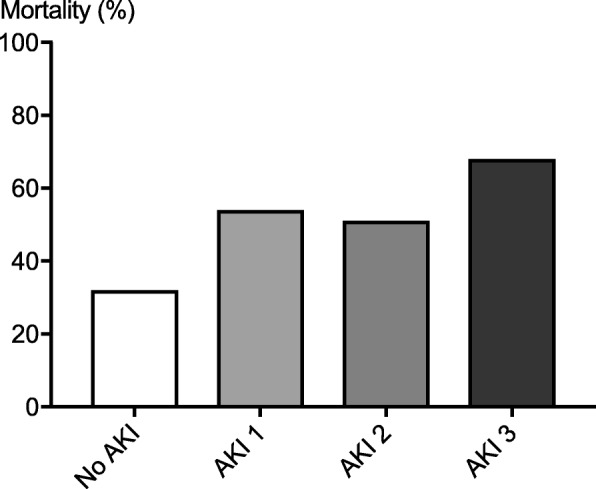
The mortality in relation to the worst stage of AKI. The worst AKI stage during the first 7 days of intensive care stay vs mortality at 6 months. The mortality was significantly higher in patients with AKI (*p* < 0.001)

Since only patients surviving beyond day 2 were included in the study, overall 6-month mortality was relatively low at 44%. In patients with AKI stage 3, 6-month mortality was significantly higher at 68% compared to 33% in patients without AKI (Fig. 4). Nine of 17 patients who were still RRT-dependent 7 days after the CA were alive at 6 months with none of them requiring long-term dialysis.

## Discussion

In survivors of OHCA from the presumed cardiac cause, temperature management at 33 °C did not show different rates of AKI from management at 36 °C. In addition, emergency contrast angiography within 6 h of OHCA was not associated with a higher risk of AKI compared to later or no angiography. This finding is important and should be evaluated in properly designed trials given the possibility that the number of patients managed with early angiography may increase if the recommended clinical strategy for STEMI is extended to non-STEMI.

Reported incidences of AKI after CA range from 12 to 81% depending on the definition used [1, 4]. Our finding of an incidence of AKI of 45% is in line with previous work [5, 19]. We also show an association between AKI after OHCA and increased mortality, which again is in agreement with previous reports [1, 2, 5, 19–21].

The TTM-33 group contained more patients with AKI and more severe AKI than the TTM-36 group. The temperature groups were reasonably well balanced with regard to patient and cardiac arrest factors. We believe the exclusion of patients who died on days 1 and 2 after OHCA from our analysis did not affect our conclusions since death in the first 2 days was evenly distributed between temperature allocations. The patient characteristics were also balanced with respect to the patient and cardiac arrest factors. During the ICU stay, vasoactive drugs were used more frequently in the TTM-33 group compared to the TTM-36 group. This may reflect differences in cardiovascular performance between the groups. A previous sub-study of the TTM trial including 171 patients showed that patients randomised to TTM-33 had a reduced cardiac index and increased systemic vascular resistance compared to TTM-36 [22]. The optimal blood pressure/perfusion parameters for renal recovery after cardiac arrest are not known. In well-characterised patients with vasoplegia, an increase in MAP from 60 to 75 mmHg led to an increase in glomerular filtration rate (GFR) and Cr-EDTA clearance without further improvement with an increase to 90 mmHg [23, 24]. In OHCA patients, there is an association not only between increasing MAP and improved renal function indices, but also between increasing level of vasoactive support and decreased GFR/increased need for RRT [25]. Considering CA patients and renal function, the optimal balance between MAP and vasoactive support is not known. Patients in the TTM-33 group also received more intravenous fluids than the TTM-36 group. Fluid overload is a risk factor for development and progression of AKI [26, 27]. This aspect of clinical management after CA deserves further attention in future studies. After correction for important risk factors, temperature allocation was no longer an independent risk factor for AKI.

After correction for important risk factors, early angiography was not independently associated with an increased risk of AKI. This is encouraging, as fear of contrast-induced nephrotoxicity may lead clinical teams to withhold potentially life-saving emergency coronary angiography unnecessarily. Similar results were noted by Petek et al. [28]. Whether the TTM protocol recommendation to administer intravenous fluids on induction of TTM (30 ml/kg) modified the development of AKI is unclear.

Our principal reason for focusing on the effects of early angiography (within 6 h of CA) was to investigate a clinical scenario in which the renal system is exposed to several potentially harmful insults simultaneously. In the post CA setting, where cardiac output may be reduced, administration of contrast during emergency angiography might potentially compromise the chances of renal recovery further [29]. The lack of an increased incidence of AKI in this setting is reassuring especially in light of current guidelines [12] and consensus statements [30, 31]. Early angiography may result in faster recovery of cardiac function and better renal perfusion, which may outweigh the potential risks associated with contrast media. Our results are supported by a recent study showing no significant difference in the release of early AKI biomarkers in critically ill patients after contrast exposure [32].

Both patient characteristics and OHCA factors were associated with development of AKI. An increasing time from CA to ROSC, an indicator of ischaemic burden, was independently associated with a higher risk of AKI, as was a higher serum creatinine on admission. The association of the intensity of ischaemia/reperfusion injury after CA (whether assessed as amount of epinephrine administered, time to ROSC, lactate concentration or the early presence of shock) and AKI has been noted in several studies [3–5, 19, 21]. Increased serum creatinine concentrations on admission and risk of AKI are also well described [4, 5, 19]. Thus, creatinine concentrations have been incorporated in most AKI risk-scoring systems [33]. The reason for the increased baseline creatinine concentration in our patient group developing AKI is not known. Patient factors (age, pre-existing cardiac failure, higher body mass index with associated muscle mass and unrecognised CKD (chronic kidney disease) and peri-arrest factors (longer time to ROSC and more pronounced post ROSC haemodynamic instability) may have contributed [34].

The presence of an IABP was strongly associated with the development of AKI, but shock and poor lactate clearance were not. Since the use of IABPs was unevenly distributed between centres, availability and indications for its application may have differed. It is possible that the use of IABP is a surrogate marker of continued haemodynamic instability and continued shock, but it is also possible that using an IABP after OHCA increases the risk of AKI per se.

Early angiography was not independently associated with a lower incidence of AKI. When extending the analysis to all patients with angiography during the ICU stay, angiography was independently associated with a reduction of AKI but the selection and survivor bias contributes to this finding.

### Limitations

It is important to acknowledge limitations to this post hoc analysis based on a randomised controlled trial designed primarily to address the postulated protective effect of temperature control after OHCA, and therefore, the results should be regarded as hypothesis generating. Data on pre-existing renal function were not available. We elected not to use the MDRD formula to assess baseline creatinine since race (black/non-black) was unknown. The use of the first recorded serum creatinine concentration on arrival to the hospital might have underestimated baseline renal function resulting in a reduced incidence of AKI [1, 7]. However, we note that it takes 24–36 h for serum creatinine changes to occur after a definite renal insult [35]. Therefore, it is likely that the creatinine values within the immediate period after OHCA are reasonably representative of pre-existing values, but it is conceivable that we missed a certain degree of unrecognised CKD. Since only daily urine output data were available, the urinary criteria of the KDIGO classification of AKI had to be modified, an approach commonly taken in large cohort studies [36]. The consequence of this modification was likely to be a reduced recorded incidence of AKI. Urine output may have been modified by for instance initial volume loading, cold-induced diuresis or use of diuretics; however, on day 2, the first complete day included in the study, there was no difference in urine output between TTM-33 and TTM-36.

Since the TTM-study was not randomised by timing of angiography, there may be a selection bias towards early angiography in relation to the first analysed rhythm (ventricular fibrillation/tachycardia) and ECG findings (ST-elevation) reflecting resuscitation guidelines. Haemodynamic instability and cardiogenic shock may have played a less prominent role in a similar selection bias because early angiography was performed with similar frequency in those patients excluded due to early death and those included. Early angiography seems to have been employed equally despite more pronounced lactate elevation and cardiogenic shock in the excluded group.

We cannot exclude that the use of early angiography was influenced by the initial creatinine concentration as the initial creatinine was lower in the early angiography group. However, the patients excluded due to early death had higher baseline creatinine than the included patients. Despite this, early angiography was applied with similar frequency. The necessary exclusion of 73 patients (8%) dying before the end of day 2 after CA may have produced an unknown bias.

We cannot exclude that some patients in our study may have been exposed to other early contrast examinations, for instance to exclude a pulmonary embolism. Finally, the TTM trial data did not include the type or volume of contrast administered including during coronary angiography.

## Conclusions

In survivors of OHCA from the presumed cardiac cause, temperature management at 33 °C did not show different rates of AKI from management at 36 °C. Coronary angiography within 6 h of CA was not associated with an increased incidence of acute kidney injury compared to later or no coronary angiography. The additional effect of coronary angiography on kidney function after cardiac arrest was negligible compared to other factors causing acute kidney injury after cardiac arrest.

The present results suggest that coronary angiography should not be deferred in a patient after cardiac arrest due to concerns over the risk of acute kidney injury.

## Additional file

Additional file 1: **Table S1.** Definitions of AKI stage as used in the study. **Figure S1.** Flow sheet showing the number of patients enrolled in the TTM trial and included in the post hoc sub-study. **Figure S2.** Number of patients and stage of AKI from days 2–7 after cardiac arrest. The declining number of patients in the latter part of the week was predominantly due to patients leaving ICU due to death, step down from intensive care as part of a treatment limitation plan or recovery. ** Table S2.** Patients stratified according to temperature allocation. Abbreviations: CHF, chronic heart failure; IHD, ischaemic heart disease; PCI, percutaneous coronary intervention; AMI, acute myocardial infarction; CABG, coronary artery bypass grafting; CPR, cardiopulmonary resuscitation; CA-ROSC, time from cardiac arrest to return of spontaneous circulation; IABP, intra-aortic balloon pump; TTM, targeted temperature management; RRT, renal replacement therapy. (DOCX 197 kb)

## Abbreviations

AKI: Acute kidney injury
BP: Blood pressure
CA: Cardiac arrest
CABG: Coronary artery bypass grafting
CHF: Chronic heart failure
CKD: Chronic kidney disease
CPR: Cardiopulmonary resuscitation
DA: Dopamine
Epi: Adrenaline/epinephrine
GFR: Glomerular filtration rate
IABP: Intra-aortic balloon pump
ICU: Intensive care unit
IHD: Ischaemic heart disease
IQR: Interquartile range
KDIGO: Kidney disease improving global outcomes
MAP: Mean arterial pressure
NA: Noradrenaline/norepinephrine
NYHA: New York Heart Association
OHCA: Out-of-hospital cardiac arrest
PCI: Percutaneous coronary intervention
ROSC: Return of spontaneous circulation
RRT: Renal replacement therapy
SOFA: Sequential Organ Failure Assessment
STEMI: ST-Elevation myocardial infarction
TTM: Target temperature management
