# Supplementary material for: Renal function after out-of-hospital cardiac arrest; the influence of temperature management and coronary angiography, a post hoc study of the target temperature management trial
Source: Crit Care. 2019 May 8;23:163. doi: 10.1186/s13054-019-2390-0 (PMC6506949; doi:10.1186/s13054-019-2390-0)
Supplement: Supplementary file 1 — Table S1. Definitions of AKI stage as used in the study. Figure S1. Flow sheet showing the number of patients enrolled in the TTM trial and included in the post hoc sub-study. Figure S2. Number of patients and stage of AKI from days 2–7 after cardiac arrest. The declining number of patients in the latter part of the week was predominantly due to patients leaving ICU due to death, step down from intensive care as part of a treatment limitation plan or recovery. Table S2. Patients stratified according to temperature allocation. Abbreviations: CHF, chronic heart failure; IHD, ischaemic heart disease; PCI, percutaneous coronary intervention; AMI, acute myocardial infarction; CABG, coronary artery bypass grafting; CPR, cardiopulmonary resuscitation; CA-ROSC, time from cardiac arrest to return of spontaneous circulation; IABP, intra-aortic balloon pump; TTM, targeted temperature management; RRT, renal replacement therapy. (DOCX 197 kb) [file 13054_2019_2390_MOESM1_ESM.docx]

**Additional file 1**

**Figure S1.** Flow sheet


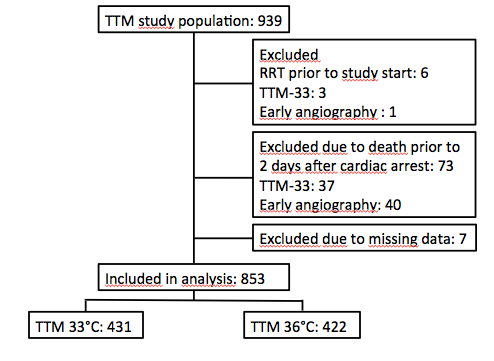


**Figure S2.** Number of patients and stage of AKI from day 2-7 after cardiac arrest.

**Table S1**. Definitions of AKI stage as used in the study.

| **Stage** | **S-Creatinine** | **Urine output** |
| --- | --- | --- |
| 1 | 1.5-1.9x baseline (within 7d) or  ≥0.3 mg/dL (≥26.5 μmol/L) increase during 48 h | Not applicable |
| 2 | 2.0-2.9x baseline | <0.5 ml/kg/h for 24 h |
| 3 | 3.0x baseline or  Increase in S-Creatinine to ≥4.0 (≥353.6 μmol/L) or  Renal replacement therapy | <0.3 ml/kg/h for 24 h |

**Table S2.** Patient data stratified according to temperature allocation.

|  | TTM 33°C (n=431) | TTM 36°C (n=422) | p |
| --- | --- | --- | --- |
| Baseline variables |  |  |  |
| Age (years) | 65 (57-72) | 65 (56-73) | 0.71 |
| Sex (male) | 361/431 (84 %) | 336/422 (80 %) | 0.14 |
| Hypertension | 176/430 (41 %) | 165/420 (39 %) | 0.58 |
| CHF (NYHA 3-4) | 29/429 (7 %) | 27/422 (6 %) | 0.89 |
| Any IHD, PCI, AMI, CABG | 147/431 (34 %) | 120/419 (29 %) | 0.07 |
| Diabetes | 53/428 (12 %) | 68/420 (16 %) | 0.12 |
| Cardiac arrest variables |  |  |  |
| Shockable rhythm | 343/431 (80 %) | 343/422 (81 %) | 0.61 |
| Bystander CPR | 318/431 (74 %) | 307/422 (73%) | 0.70 |
| Time CA-ROSC (min) | 25 (17-37) | 25 (15-39) | 0.55 |
| Shock on admission | 56/431 (13 %) | 56/422 (13 %) | 1.00 |
| Lactate on admission (mmol/L | 5.6 (2.8-9.0) | 5.3 (2.4-8.8) | 0.77 |
| IABP | 76/431 (18 %) | 68/423 (17 %) | 0.58 |
| Angiography within 6h of CA | 262/424 (62 %) | 254/411 (62 %) | 0.94 |
| Any vasoactive drug |  |  |  |
| Day 1 | 329/427 (77 %) | 320/418 (77 %) | 0.87 |
| Day 2 | 373/425 (88 %) | 332/418 (79 %) | 0.001 |
| Day 3 | 338/425 (80 %) | 274/418 (66 %) | <0.001 |
| Noradrenaline or Adrenaline >0.1 μg/kg/min d 1-3 | 242/431 (56 %) | 199/422 (47 %) | 0.45 |
| Renal variables/outcomes |  |  |  |
| Baseline creatinine (μmol/L) | 105 (85-125) | 105 (85-125) | 0.12 |
| Creatinine >130 μmol/L | 73/420 (17 %) | 77/412 (19 %) | 0.65 |
| Worst AKI stage in first week |  |  | 0.018 |
| stage 1 | 78/431 (18 %) | 60/422 (14 %) | 0.14 |
| stage 2 | 63/431 (15 %) | 53/422 (13 %) | 0.42 |
| stage 3 | 70/431 (16 %) | 57/422 (14 %) | 0.29 |
| Any AKI first week | 211/431 (49 %) | 170/422 (40 %) | 0.01 |
| Daily fluid balance |  |  |  |
| Day 2 | 1700 (675-2925) | 1300 (375-2200) | <0.001 |
| Day 3 | 500 (-400-+1700) | 300 (-600-+1200) | 0.001 |
| RRT during first week | 38/432 (9 %) | 36/422 (8 %) | 0.90 |
| Survival at 6 months | 238/431 (55 %) | 236/422 (56 %) | 0.78 |
